# Including ultrasound scans in antenatal care in low-resource settings: Considering the complementarity of obstetric ultrasound screening and maternity waiting homes in strengthening referral systems in low-resource, rural settings

**DOI:** 10.1053/j.semperi.2019.03.017

**Published:** 2019-08

**Authors:** David L. Swanson, Holly L. Franklin, Jonathan O. Swanson, Robert L. Goldenberg, Elizabeth M. McClure, Waseem Mirza, David Muyodi, Lester Figueroa, Nicole Goldsmith, Nancy Kanaiza, Farnaz Naqvi, Irma Sayury Pineda, Walter López-Gomez, Dorothy Hamsumonde, Victor Lokomba Bolamba, Jamie E. Newman, Elizabeth V. Fogleman, Sarah Saleem, Fabian Esamai, Sherri Bucher, Edward A. Liechty, Ana L. Garces, Nancy F. Krebs, K. Michael Hambidge, Elwyn Chomba, Melissa Bauserman, Musaku Mwenechanya, Waldemar A. Carlo, Antoinette Tshefu, Adrien Lokangaka, Carl L. Bose, Robert O. Nathan

**Affiliations:** a University of Washington, Seattle, WA, United States; b RTI International, Research Triangle Park, NC, United States; c Columbia University, New York, NY, United States; d Aga Khan University, Karachi, Pakistan; e Moi University, Eldoret, Kenya; f INCAP, Guatemala City, Guatemala; g University of Zambia, Lusaka, Zambia; h Kinshasa School of Public Health, Kinshasa, Democratic Republic of the Congo; i University of Colorado, Denver, CO, United States; j Indiana University, Indianapolis, IN, United States; k University of Alabama at Birmingham, Birmingham, AL, United States; l University of North Carolina at Chapel Hill, Chapel Hill, NC, United States

**Keywords:** Pregnancy risk screening, Maternity waiting home, Referral systems, Continuum of care, Task shifting, Midwifery

## Abstract

Recent World Health Organization (WHO) antenatal care recommendations include an ultrasound scan as a part of routine antenatal care. The First Look Study, referenced in the WHO recommendation, subsequently shows that the routine use of ultrasound during antenatal care in rural, low-income settings did not improve maternal, fetal or neonatal mortality, nor did it increase women's use of antenatal care or the rate of hospital births. This article reviews the First Look Study, reconsidering the assumptions upon which it was built in light of these results, a supplemental descriptive study of interviews with patients and sonographers that participated in the First Look study intervention, and a review of the literature. Two themes surface from this review. The first is that focused emphasis on building the pregnancy risk screening skills of rural primary health care personnel may not lead to adaptations in referral hospital processes that could benefit the patient accordingly. The second is that agency to improve the quality of patient reception at referral hospitals may need to be manufactured for obstetric ultrasound screening, or remote pregnancy risk screening more generally, to have the desired impact. Stemming from the literature, this article goes on to examine the potential for complementarity between obstetric ultrasound screening and another approach encouraged by the WHO, the maternity waiting home. Each approach may address existing shortcomings in how the other is currently understood. This paper concludes by proposing a path toward developing and testing such a hybrid approach.

## Introduction

As of 2016, the World Health Organization (WHO) recommends an ultrasound scan as a part of routine antenatal care (ANC). This recommendation is part of a comprehensive WHO guideline on routine ANC for pregnant women and adolescent girls.^1^ Specifically, one ultrasound scan before 24 weeks’ gestation is recommended for pregnant women to estimate gestational age, improve detection of fetal anomalies and multiple pregnancies, reduce induction of labor for post-term pregnancy, and improve a woman's pregnancy experience.

The development group for the WHO recommendations acknowledges that the use of early pregnancy ultrasound has not been shown to reduce perinatal mortality. The Global Network First Look Study (2013–2016), which was underway at the time of publication, is discussed among the considerations of the ultrasound recommendation. This multi-country cluster randomized trial, according to the WHO ANC guideline, “should contribute further evidence on the health effects, health care utilization and implementation-related information on ultrasound in rural low-resource settings.^1^ ”

Of these expectations, the First Look Study did provide further evidence on health effects and health care utilization when the results were published in 2018. The results showed, however, that the routine use of ultrasound during ANC did not improve maternal, fetal or neonatal mortality, or maternal near-miss. Moreover, ultrasound screening during ANC did not increase women's use of ANC or the rate of hospital births.^2^

The expectation that the study should generate ‘implementation related information’ on ultrasound in low-resource settings was also met. Elements of the study's implementation have been discussed in detail elsewhere, including the training of ultrasound naïve health personnel in obstetric ultrasound screening,^3^ the development of web-based quality assurance,^4^ and an evaluation of their combined effectiveness and accuracy.^5^ A subsequent case study on challenges of implementing the obstetric ultrasound screening for the First Look Study in the Democratic Republic of the Congo details some of the political, logistical, infrastructural and resource challenges acknowledged in the WHO guideline.^6^

This article reviews the intervention of the First Look Study, reconsidering the assumptions upon which it was built in light of the study's results, WHO recommendations, a supplemental study of interviews with patients and sonographers that participated in the First Look Study intervention, and a review of the literature. What surfaces is a potential for complementarity between obstetric ultrasound screening and another approach encouraged by the WHO, the maternity waiting home.^7^ Each approach may address existing shortcomings in how the other is currently understood. A hybrid approach might also strike a better balance between evaluation and implementation. This paper concludes by proposing a path toward developing and testing such a hybrid approach.

## The First Look Study and the WHO ultrasound recommendation

The First Look Study was a cluster-randomized trial conducted in rural areas of the Democratic Republic of Congo (DRC), Guatemala, Kenya, Pakistan, and Zambia primarily to evaluate the impact of obstetric ultrasound screening at routine ANC visits on maternal, fetal and neonatal mortality and maternal near-miss. The study aimed, secondarily, at evaluating the intervention's effect on women's use of ANC and the rate of hospital births. The study also assessed the quality of the field sonographers’ ultrasound examination through review of a proportion of the examinations by expert sonographers using a web-based program.^4^ Finally, First Look investigators conducted a qualitative study to better understand the reasons for women's acceptance of referrals.

Clusters were predefined geographical areas with a health center, approximately 300–500 expected deliveries per year, and a Maternal Newborn Health Registry, an independent study which documented all pregnancies and their outcomes to 6 weeks post-delivery. Ultrasound units and training were introduced in intervention cluster health centers, as well as in those hospitals to which patients from intervention and control clusters were referred. Standard care was provided in the control clusters.

The trial was approved by all participating institutional review boards and ethic review committees; all women and sonographers who participated provided informed consent. The study design, training, and ethical approvals are described in detail elsewhere.^3^, ^8^

### Task shifting

The recent WHO ANC guideline notes that antenatal ultrasound is a task which potentially can be shifted from trained sonographers and doctors at hospitals to skilled attendants in rural health care facilities.^1^ This approach was utilized in the First Look Study, where obstetric ultrasound screening during ANC visits was performed by rural health center personnel. We use the term ‘field sonographer’ throughout to encompass the skilled attendants – the nurses, midwives, medical and clinical officers – trained to use ultrasound at ANC in the First Look Study intervention clusters. Field sonographers were taught to assess gestational age, to identify high-risk pregnancies – including multiple gestations, malpresentation, placenta previa, intrauterine growth restriction, and some fetal anomalies – and when and how to refer patients to hospitals providing comprehensive emergency obstetric and neonatal care.

The WHO ANC guideline also emphasizes the importance of quality assurance, ongoing training, supervision and staff retention, all of which were reinforced by the First Look Study. The concept of ‘obstetric ultrasound screening’ employed in the study embodies the integration of task-shifting with oversight. In this approach, positive screening results require confirmation before consequent interventions are undertaken. Patients screening positively for high-risk pregnancies by field sonographers at intervention health centers, for example, were encouraged to refer to a hospital for a confirmatory ultrasound by hospital sonographers and physicians. This relationship, with the field sonographer acting as an extension of the hospital sonographer, also provided supervision. With the assistance of a web-based quality assurance process, involving the remote review of stored images of ultrasound scans, hospital sonographers could be aimed or could target where continued training of field sonographers in obstetric ultrasound screening was required.^4^ Hospital sonographers also trained replacement field sonographers in obstetric ultrasound screening as necessary. A strong indication of the effectiveness of these task-shifting and quality assurance processes is seen in the review of stored images using the web-based quality assurance process. The concordance between field sonographers and reviewers in the ultrasound diagnosis was 99.4%.^5^

### The continuum of care

Through a broader lens, the First Look Study's intervention increased the pregnancy risk screening capacity of skilled attendants at rural, primary health care facilities in low-resource settings. The effectiveness of skilled attendants on improving healthy pregnancy outcomes in these settings, according to the WHO, is also dependent upon their role within a continuum of care.^9^ For the sake of clarity, ‘continuum of care’ in this article refers to the household-to-hospital continuum of care, spanning the home, community, health center, and hospital.^10^, ^11^ The continuum starts at home with the woman and her family, is followed by first level care that involves the provision of high-quality care, and – when complications occur – may require care at secondary or tertiary levels of the health system. The importance of a viable continuum of care in ensuring quality care at the time of birth remains a priority in recent literature.^12^

An awareness of the importance of this continuum of care is apparent in the First Look Study's protocol.^8^ Although the focus of the study's intervention was on training skilled attendants, other inputs were incorporated across the continuum. Skilled attendants were trained to be field sonographers, for example, and to encourage women screening positively for potential complications to refer to hospitals. Hospital sonographers were taught to confirm the findings of these screenings upon referral. In communities targeted for the intervention, sensitization activities were conducted to inform women and their families of the availability of ultrasound at their antenatal care clinics. In referral hospitals, training in the management of obstetric and neonatal care was provided to staff as necessary. Furthermore, guidance was offered to health system officials and hospital administrators on possible referral system enhancements. An outward manifestation of this guidance was the creation of referral algorithms – developed with input from the local health system and posted near each ultrasound – to help field sonographers determine the need for and the timing of referrals.

Increasing the capacity of the skilled attendant, however, remained the primary focus of the study's intervention. As such, the First Look Study depended in large part upon the skilled attendant playing a central role in the continuum of care. The training emphasized the importance of communicating with the patient during the scan, helping the patient understand what ultrasound can show, and incorporating these findings into a patient's birth-preparedness plan. These emphases correspond to the skilled attendant's ‘core skills and abilities’ spelled out by the WHO, including the capacity to “assess individual needs, give appropriate advice and guidance, calculate the expected date of delivery and perform specific screening tests.^9^” The skilled attendant's enhanced risk-assessment capabilities and effectiveness in using them to encourage patients with high-risk pregnancies to deliver at a hospital, in other words, were the fundamental means by which pregnancy outcomes were intended to improve.

This concept of positioning the skilled attendant at the center of the continuum of care is one that the WHO has strongly advocated. This concept is articulated in the 2004 joint statement of the WHO, the International Confederation of Midwives and the International Federation of Gynecology and Obstetrics, *Making pregnancy safer: the critical role of the skilled attendant*. The statement emphasizes that in childbearing, “women need a continuum of care to ensure the best possible health outcome for them and their newborns.” In doing so, it positions the “skilled attendant… at the center of this continuum of care.” It elaborates on this, stating, “At the primary health care level, she/he will need to work with other care providers in the community, such as traditional birth attendants and social workers. She/he will also need strong working links with health care providers at the secondary and tertiary levels of the health system.^9^ ”

The results of the First Look Study, showing no improvement in maternal, fetal and neonatal outcomes and no increase in the rate of hospital births, however, may open the possibility of revisiting the reasoning behind this approach. Perhaps, even with enhanced risk-assessment and improved birth-preparedness skills, the position of the skilled attendant – particularly one stationed in a rural health center in a low-recourse health system – lacks the agency to improve and maintain a continuum of care sufficient for improved outcomes.

## The First Look Study and descriptive study

### Communicating across the continuum of care

Because the First Look Study was conducted across 5 study sites in 5 different countries, numerous concerns arose; some unique, some ubiquitous.^6^ Approaches to addressing these concerns were shared across sites at the study's inception and throughout its course. Bound by the study's protocol and respectful of the autonomy of each country site within the Global Network, solutions implemented in one site were suggested to other sites. Many of these suggestions were aimed at improving the continuum of care through small measures which incorporated ultrasound-enhanced risk-screening.

An approach originating from the Kenya site involved the use of a black book that tracked those patients with potentially high-risk pregnancies. A patient, for example, with a fetus lying transversely, was recommended to return to the health center for a follow-up scan at 36 weeks to determine whether the condition persisted and referral was recommended. The black book contained the recommended date of the follow-up scan and a contact cellphone number of the patient, a family member, a community health worker or a neighbor, depending upon availability. A call was placed to encourage a follow-up visit by the patient once the recommended date passed. This simple approach aimed to strengthen the home-to-health center section of the continuum of care. The prevalence of cellphone technology makes this possible and would be enhanced by well-organized community health worker networks.

Across study sites, a similar system was suggested for the hospitals to which patients from intervention health centers were referred. Here hospital staff – often sonographers – were encouraged to receive calls or texts from field sonographers and record the screening results and timing of referrals. In the ideal, a hospital could then keep track of expected referrals, communicating back to health centers when an expected patient did not arrive by a given date. Personnel at the health center could then reach out across the continuum of care to the community and household to further encourage the patient to make her referral.

Where First Look Study sites encouraged field sonographers to communicate at the household and community levels, real and perceived hierarchies in health systems made field sonographers communication with referral hospitals substantially more problematic. This is reflected in interviews conducted as part of a descriptive study in the intervention clusters of the First Look study, described in detail elsewhere.^13^ The descriptive study consisted of structured interviews conducted near the end of the 18-month study at all five country sites. Individual structured interviews were conducted in each site with field sonographers, hospital sonographers, and patients recommended for referral during ANC ultrasound screenings.

In these structured interviews, 3 of the 38 field sonographers responded – when asked for “any other comments regarding the ultrasound referral” – with basic concerns about communication with referral hospitals. Following are their responses: “Need of a direct contact person at the referral centre;” “Proper link to referral site like phone numbers and the person receiving a client on the other side;” “Patients are not told who will attend them.” When asked for some of the reasons that women are not going to the referral visit, 8 of 38 field sonographers specified that “The referral hospital is not attentive to patients that we send.” Field sonographers’ responses were channeled to boxes checked by the interviewers, and multiple boxes could be checked, the last being “Other, specify.” Two responses specified: “Non-availability of the referral sonographer and the woman gave birth before the sonographer was available” and “Don't know which department to go to as where (sic) it was located.” Details of the survey are described elsewhere.^13^

Where the field sonographers could communicate with the hospital about referrals, they appear not to have had the means to ensure that their improved risk-assessment capacity was used effectively. Even where they had “strong working links” with hospital health care providers, as recommended by the WHO,^9^ these seemed insufficient to bring about the structural change necessary to accommodate their improved risk screening capacity. In Zambia, for example, the relationship between field sonographer and hospital sonographer was established through two weeks of initial training and was strengthened through continued training and communication over referrals. As a result, the hospital sonographer received referred patients directly, saving patients from ANC processing at the hospital, which could consume a day. The sonographer could not, however, facilitate the patient's interaction with the hospital further than the provision of a confirmatory scan. In a structured interview with hospital sonographers conducted concurrently with those mentioned above, one sonographer responded as follows when asked at the end of a structured survey interview if there is anything else he thought important to mention: “The rest of (the hospital) is not as responsive or say they have no bed space (or) more serious conditions to deal with.” Another respondent mentioned, “If the (maternal child health department) was better engaged,” similarly seeming to desire better connectivity across hospital departments.

Concerns surrounding the need for structural change in hospitals to accommodate the improved risk screening of the intervention surfaced in all study sites throughout the course of the study.^6^ This observation suggests that the study's emphasis on training skilled attendants, in line with WHO recommendations, with merely the provision of guidance to health system and hospital officials, may in retrospect have been insufficient to improve outcomes. The temporary nature of the study and the need to contain its intervention into a measurable, scalable entity may have limited the intervention's impact as well.^6^ The importance of making structural changes to referral systems, particularly along the health center-to-hospital section of the continuum of care, to accommodate the increased capacity in risk screening of ultrasound rose to prominence for some investigators during the course of the study. In light of the study's results, this aspect of the approach appears to warrant further scrutiny.

### The complexity of referral

Obstetric referrals from rural primary health centers to hospitals in low-resource settings involve levels of complexity that can create barriers for rural women. This receives little to no attention in the literature concerned with ultrasound in ANC in low-resource settings. Moreover, the task-shifting nature of obstetric ultrasound screening, with the corresponding need for confirmation, may increase this complexity.

To better understand this complexity, consider, as an example, a woman from Lukolis, a rural community in Kenya. Upon receiving an ultrasound scan as a part of an antenatal visit at her local health center, this woman is told by the field sonographer that she has screened positively for twins. The field sonographer recommends the woman refer to a hospital in the city of Busia, 20 km away, for a confirmatory ultrasound, ideally initiating a process intending the woman to deliver there.

In the literature concerning ultrasound in antenatal care, a referral is often considered a kind of finality for the ultrasound intervention. The field sonographer discovers potentially complicated pregnancies and refers those patients for treatment at the referral hospital, theoretically leading to better maternal and neonatal outcomes. This is expressed in terms like: “Once diagnosed, patients with complicating conditions…would ideally be referred to a regional obstetric center where they would be managed appropriately.^14^ ”

The First Look Study protocol reflects the literature in this regard. The protocol emphasized that for obstetric ultrasound screening in primary health centers to have a chance at being effective: “Having a referral institution with staff trained to review ultrasound findings and manage complications is crucial.^8^ ” As to what happens to the patient between being referred at the primary health center and delivering at the hospital, the protocol is brief, limiting the extent of intervention to be targeted at this section of the continuum of care. In its discussion of referral and system enhancement the protocol says, “While this will not be a major trial component, we expect to hold several sessions with appropriate health system leaders and administrators to discuss integration of obstetric/neonatal care between the primary health clinics and referral hospitals.^8^ ” Again, this was because the focus was on building the capacity of the skilled attendant who, in the words of the WHO, is “pivotal in reducing maternal mortality and morbidity.^9^ ”

What appears to be overlooked here is the complexity of a referral from the patient's perspective. For the woman from Lukolis, Kenya, a referral means that she must pay for a ride on a minibus to the district hospital in Busia. Once she finds the hospital, she discovers that she must first attend a processing visit in the ANC department before she can schedule a confirmatory ultrasound scan in the radiology department. It takes most of the day for the ANC visit to be completed, and the confirmatory scan is scheduled for the following morning. She now either needs to travel back home and return to Busia Hospital by the morning or find a place to spend the night in Busia.

This complexity is reflected in the patients’ responses during interviews conducted as part of a descriptive study mentioned above.^13^ Individual structured interviews for patients were conducted in each country site with a convenience sample of women at 6 weeks post-delivery for whom referral was recommended during ANC ultrasound screenings in primary health centers. An additional interview was conducted with women who made the referral; another for those who did not. Of the 190 interviews conducted with women who did not make their referrals, 54 indicated that they attempted to visit the referral hospital. Table 1 compiles the reasons that women did not attend the referral visit. Of these responses, 25 pertain to the patient not receiving adequate attention at the hospital and 13 relate to the hospital being an intimidating and/or difficult place for a patient to find her way through.

**Table 1:** Reasons women did not attend the referral visit.

Reason	Frequency
I was told to come back later	14
I did not know where to go in the hospital	10
I was not attended to on the day I visited	5
I was not comfortable being at the hospital	3
I was told to come back the following day and had nowhere to spend the night	3
I had an appointment, but was not attended to on that day	3
Multiple response	10
Missing	6

Of the 135 women interviewed who *did not attempt* to visit the referral hospital, the barriers most often identified were cost (45% of interviewees), transportation (16%) and distance to the hospital (14%). Disapproval by the father (20%), other family members/neighbors (9%), and traditional healers or clergy/pastor (7%) were also among the barriers cited. Concerns about the hospital – ‘heard about bad experiences as the hospital’ (10%), ‘not comfortable going to the hospital’ (7%) – also surfaced in the responses. 10 interviewees (7%) who responded to an “Other, specify” option also cited concerns with the hospital, including, “hospital staff treats patients poorly” and “fear of the hospital.”

Continuing with our example, suppose the woman from Lukolis overcomes these initial barriers, traveling 20 km home for the night and returning to Busia District Hospital the next day. There the sonographer is able conduct an ultrasound exam confirming that she has twins. The woman is then told to go to the maternity ward. The nurses there inform her that she should deliver at the hospital; but, because of the limited availability of beds, she should present at the hospital only once labor begins. The woman now needs to find and afford direct transportation from her home in Lukolis or stay near the hospital in Busia in the days or weeks before her due date, allowing her to be present at the hospital soon after the onset of labor.

Instead of a simple visit to the hospital, the woman's referral now requires three visits to Busia and possibly accommodation in the city for days or weeks. Each of these steps increases costs and time away from a household that depends upon her. Each step represents an additional barrier which may keep the woman from delivering in a facility that provides the comprehensive emergency obstetric and neonatal care she may need.

The structured interviews conducted in conjunction with the First Look Study provide a glimpse into how women are affected by additional barriers created by having to travel to the referral hospital more than once. Of the 510 women that indicated that they attended a referral visit during their pregnancies when asked at 6 weeks post-delivery, 121 indicated that they did not deliver at a hospital. Table 2 details reasons women provided for why they did not deliver at a hospital. The additional costs in terms of time and money of returning to the hospital at delivery became prohibitive for many.

**Table 2:** Reasons why women that attended the referral visit did not deliver at a hospital.

Reason	Frequency	Percent
Expense / lack of money	21	19.63%
Time	23	21.50%
No transportation	10	9.35%
Distance to referral hospital	13	12.15%
Other, specify	40	37.38%
Frequency missing = 14		

Of those who responded “Other”, 9 responses related to the timing of deliveries in the vein of: “labour was sudden” or “delivered before time.” 9 responses referred to matters of choice like: “there was no need, the baby was fine” or “she was afraid to have a cesarean,” while another 4 indicated that delivering at the health center seemed sufficient with statements such as: “Midwife at the clinic was able to deliver the mother.” 5 responses refer to advice or a change in diagnosis such as: “In the hospital they told me that everything was fine” or “Baby changed position to normal.” 6 responses specified concerns with the hospital such as: “They do not take good care at the hospital” or, perhaps most notably, “I went to deliver at the hospital, but it took long for baby to come, I was sent back home. At arrival in my residence the labor worsen after 20 min I delivered my baby boy and girl.” The remaining responses indicated some misunderstanding of the question, of the response, or in the translation.

The series of interviews referenced here was conceived by the investigators at a point during the study when concerns about the referral processes across sites mounted and preconceptions of the barriers to referral began to predominate. What these interviews provide is some elucidation into the complexity of a referral as experienced by the patient. Through this lens, the barriers relating to money, time, distance and social and cultural constraints appear to remain formidable; but, they may be compounded by the poor quality of reception that referral hospitals offer patients. Moreover, by increasing the number of visits to a referral hospital a woman is asked to make during pregnancy, obstetric ultrasound screening may to some extent counteract its intended outcome of helping more women with high risk pregnancies deliver in hospitals.

## Discussion

With the WHO ANC guideline mentioned at outset desiring the First Look Study to generate ‘implementation related information’, we turn here toward recommendations for future approaches of incorporating obstetric ultrasound screening into ANC provision in rural primary health care settings. In this regard, two themes emerge from this review. The first is that the focused emphasis that the First Look Study put on building the skills of the skilled attendant did not take into account the lack of agency rural primary health setting personnel have in building and improving the continuum of care in the direction of the referral hospital. The second is that a means of manufacturing such agency to improve the quality of patient reception at referral hospitals may be essential for obstetric ultrasound screening to have its desired impact.

Improvements on approaches to incorporating obstetric ultrasound screening into ANC, in this light, should have a dual emphasis on increasing the risk-screening capacity of health center personnel and improving the quality of reception of patients at referral hospitals. The concept of ‘streamlining’ patients who have screened positively for high-risk pregnancies, enabling them to bypass ANC processing, receive a confirmatory scan, and meet with an obstetrician or nurses in the maternity department for the purpose of developing birth-preparedness plans, all within a day's visit to the hospital, has been discussed elsewhere.^6^ Beyond streamlining, the need exists for improvements that address the barriers of transport, timing and cost for women without an immediate means of reaching the hospital at the onset of labor. In the ideal, what is needed is an entity within health systems that can build, improve and maintain continuums of care in a way less heavily reliant on health center personnel at their centers.

### Maternity waiting homes

In 1996, the WHO published *Maternity Waiting Homes: A review of experiences*, endorsing the concept as low-cost means of bringing women closer to needed obstetric care, as part of a comprehensive package of essential obstetric services.^7^ The maternity waiting home (MWH) is loosely defined as a shelter located near a hospital or primary health center for pregnant women to reside for a period prior to delivery.^15^ MWHs range from simple shelters to facilities with beds, showers, and kitchens, managed by nurses, linked to the adjoining hospitals, and offering health-related courses to visiting women by day. With encouraging anecdotal evidence indicating that MWHs were successful in reducing maternal mortality, the WHO report states in 1996 that “little quantitative research has been conducted to prove their efficacy.^7^ ” Two decades later, a Cochrane review still found insufficient evidence to determine the effectiveness of MWH for improving maternal and neonatal outcomes.^16^

The concept of prenatal risk selection has played an important role in descriptions of the MWH.^16^, ^17^ Risk screening algorithms that include maternal age, parity, height, and obstetric history – sometimes factoring in the distance of the patient's home from the health facility – have been used to determine which patients are recommended to MWHs. These have tended to have relatively low positive predictive values because of the low-risk and high-prevalence of conditions targeted in the algorithms.^18^ A frequent concern that arises in the literature is whether these risk screening algorithms lead to an effective use of resources.^16^ On the other hand, much of prenatal care is devoted to screening for specific conditions not likely to be diagnosed by the algorithms described above or by ultrasound, such as preeclampsia. The discussion below is relevant to these conditions as well.

### A combined intervention

Within the breadth of MWHs' definition may be found a complementary intervention to obstetric ultrasound screening in ANC. The WHO review emphasizes that the MWH “is not a stand-alone intervention, but rather serves to link communities with the health system in a continuum of care.^7^ ” Our findings point to the need for a strengthening of this continuum of care to make effective use of increased risk screening in remote health care settings. Determining more concise parameters and minimum requirements for what constitutes MWHs may help strengthen the continuum of care where it appears most needed.

It is worth noting that, by combining the MWH with the improved risk screening capacity of ANC ultrasound, as well as other screening tests, the result should constitute a needed improvement acknowledged throughout the literature on MWHs. With a 99.4% level of diagnostic accuracy of ultrasound screenings determined through web-based quality assurance in the First Look Study,^5^ and with the conditions discovered by ultrasound mostly low-prevalence, high-risk pregnancy complications,^18^ risk selection may become one of the combined intervention's strengths.

With regard to the themes that emerged from this review – keeping in mind scalability and measurability – we recommend some parameters for the MWH. Each of these recommendations reflect the experience of the First Look Study, the development of its ultrasound intervention, other conditions screened for during ANC, and aspects of existing MWHs described in the literature. We recommend that the following be evaluated:

1. An MWH connected to and in the vicinity of a referral hospital that provides continuous, quality comprehensive emergency obstetric and neonatal care.
2. Hospital staff engaged at an MWH to strengthen the link between communities and health systems, increasing connectivity with the referral hospital in particular.
3. An MWH tasked with managing referred patients’ relationships with the hospital, streamlining their interactions with its departments, to ensure sufficient agency exists along the continuum of care to advocate on their behalf.
4. Non-emergent, screened patients referred to an MWH, allowing the MWH to manage and track referrals and communicate with primary health centers for follow-up.
5. An MWH house and care for those screened patients required – while seeking hospital care – to spend the night away from home, as well as those with high-risk pregnancies nearing their due date or the appointed date for a planned cesarean section or induction.

Further parameters might be established through discussion with stakeholders and evaluators of MWHs, with emphasis placed on allowing for local adaptations to accommodate cultural norms and political environments.

Finally, one reason that the First Look Study of routine use of ultrasound during ANC may have failed to improve maternal, fetal or neonatal mortality could be that the conditions screened by ultrasound may not present enough risk, even if appropriately treated, to have influenced these outcomes.^2^ Some of the issues brought to light by the study and the accompanying structured interviews, however, likely pertain to remote obstetric risk screening more broadly. Patients screening positively for preeclampsia, for example, may benefit from improvements along the continuum of care that an MWH aimed at accommodating remote obstetric risk screening provides. Similarly, as improvements on risk screening are developed, this approach may provide a basis for their integration into rural, low-resource health settings.

## Conclusion

With the existing WHO recommendation of one ultrasound scan before 24 weeks’ gestation for pregnant women, and in light of the First Look Study's and its supplementary study's results, this article considers a means of improving the impact of obstetric ultrasound screening in ANC. In line with recent findings, our recommendations aim to improve access to a range of maternal health services, which has been shown to be one of the two most important predictors of maternal mortality, along with per capita income.^16^, ^19^ By combining the interventions of obstetric ultrasound screening and screening for other high-risk conditions with a redefined MWH, the possibility exists for improving and maintaining the continuum of care in a way that focusing primarily on building the capacity of the primary health center personnel appears unable to do. At present, evidence that triaging mothers with high obstetric risk for hospital delivery or to an MWH may be associated with improved perinatal outcomes remains limited to low-quality observational studies, although it is recommended that risk screening tools be evaluated as an intervention in combination with access to obstetric care.^18^ In current analyses of MWHs, ultrasound has not been considered as a tool for risk screening in rural, low-resource settings. We recommend researching the development and use of the MWH as a means of capturing the benefits of the improved risk screening which ultrasound provides. To do this, we recommend first defining more sharply the parameters of the MWH with regard to its role in supporting remote obstetric risk screening. The refined MWH could then be piloted in different settings with existing ultrasound and other screening services to better understand the impact it can have on improving communication along the continuum of care and the quality of high-risk patient referrals. If evaluations of the piloted MWHs warrant, a study on whether obstetric ultrasound screening with MWHs positively impacts maternal and neonatal outcomes might further the understanding of ultrasound's value in rural, primary antenatal care in low-resource settings.

## Ethics approval and consent to participate

The First Look Study and subsequent descriptive study were approved by all participating institutional review boards and ethic review committees; all women and sonographers who participated provided informed consent. The study design, training, and ethical approvals are described in detail here:^3^, ^8^

## Consent for publication

Not applicable.

## Availability of data and material

The data that support the findings of this study are available through the corresponding author upon reasonable request.

## Competing interests

The authors declare that they have no competing interests.

## Funding

The study was conducted by sites of the Eunice Kennedy Shriver National Institute of Child Health and Human Development (NICHD) Global Network for Women's and Children's Health Research through grants from NICHD to the sites and to RTI International, the data coordination center. Funding for the trial also was provided by the Bill and Melinda Gates Foundation: OPP1056057 to the sites with additional funding to RTI. General Electric provided the ultrasound units to the sites and in addition provided support to the Radiology Department of the University of Washington for the training component of the study. None of the funders were involved in the data analysis or writing any of the manuscripts using data from this study.

## Authors’ contributions

DS, HF, JS, EM, RG, RN, JN, CB and WC were involved in drafting the manuscript or revising it critically for important intellectual content in line with their substantial contributions to conception, design and analysis and interpretation. WM, DM. LF, NG, NK, FN, ISP, WLG, DH, VLB, EF, SS, FE, SB, EL, AG, NFK, KMH, EC, MB, MM, AT and AL made substantial contributions to conception and design, or acquisition of data, or analysis and interpretation of data.
